# A method to reproduce pH and Eh environmental changes due to sediment resuspension

**DOI:** 10.1016/j.mex.2022.101751

**Published:** 2022-06-04

**Authors:** M. C Vicente, C.L. Trevisan, A. C. B Carvalho, W.T. Machado, J.C. Wasserman

**Affiliations:** Post-Graduate Program in Geochemistry, Universidade Federal Fluminense, 24.020-141 Niterói RJ, Brazil

**Keywords:** Resuspension, Trace metals, Polluted Sediments

## Abstract

During the last decades, metals have been released into coastal areas increasing the environmental and human health risks, however, resuspension events of trace metals polluted sediment could represent even more severe risks. Anoxic condition in the sediment is capable to stabilize the trace metals, due to the bonding with reduced anions. Although, the sediment resuspension can alter the potential redox and pH characteristics resulting in metals released from the water column. The climate change advance would impact directly on ocean chemistry, is expected the spatial increase of anoxic sites, mainly in coastal areas. Furthermore, it is mandatory and urgent to expand the knowledge over the process of sediment metals releasing in order to develop prediction and remediation tools for possible environmental impacts. This is a simple method of manipulating and simulating physicochemical alterations. The creation of microcosmos without oxygen allows the formation of a very reducible environment, common in coastal areas with low energy and high organic matter input. And further oxidation allows the assessment of the trace metals released to the water column and/or the new arrangement of these metals in different geochemical fractions.

• The experimental procedure to assess trace metals mobility to potential redox and pH changes in sediment and water.

• A method is suitable for a wild range of sediment characteristics.

## Graphical abstract

**Figure:**
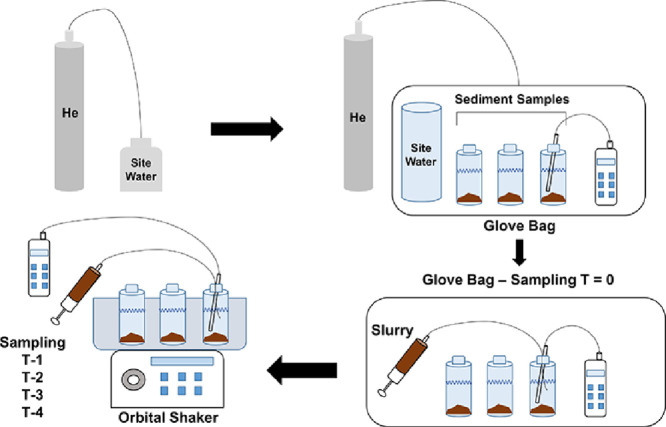

Specifications table

**Table:** 

Subject Area:	Environmental Science
More specific subject area:	*Environmental impacts for trace metals*
Method name:	*Physicochemical simulation of Eh and* pH *changes due to sediment resuspension in anoxic condition*
Name and reference of original method:	*None*
Resource availability:	*Not applicabe*

*Method details

## Overview

Sediments resuspension become an environmental issue all over the world, especially due to dredging activities. The very most of international trade is made through the oceans, resulting in periodical dredging for harbor activities maintenance, therefore sediments resuspension. The resuspension may cause disturbances to the coastal ecosystem, at the first moment increasing suspended particulate matter and photic zone narrowing [14]. However, trace metals polluted sediments could release relevant amounts of metals for water column in bioavailable chemical species, increasing the possibility of ecotoxicological events [3].

The dredge intensification related to world economic development increased the concern with possible ecosystem damages due to sediment resuspension, this encouraged research to pursue knowledge over the sediment metals release process and kinetic. In order to predict those metals release impacts, several resuspension experiments were developed during the last Decades [1, 2, [5], [6], [7], 9, 11, 13].

Most of these experiments are based on the oxidation of the sediment, in a variety of methods. Oxidation by agitation is the most common, however, other oxidation processes are also performed, such as using an air pump or pumping oxygen directly from a gas cylinder [2, 8]. Mostly related to biogeochemical studies, microcosmos is also used to simulate environmental Physico-chemical changes [4, 10]. Although, for severe redox reductive sediments the traditional oxidation methods could not be the best environmental simulation on the resuspension experiment, especially for trace metals release kinetic assessment. Furthermore, this experiment innovates reproducing the resuspension of severe redox reducing sediment conditions to assess the metals released for the water column and kinetics process among sediment geochemical fractions.

## Sediment and water sampling

For the experiment were chosen 6 points for sediment sampling, considering the possible difference between the grain size and organic matter content. The sediment was collected using a Van Veen sediment sampler. The Eh and pH environmental conditions were registered at the site using a multiparameter Hanna® probe, this procedure was performed for each sample. The samples were transported in a refrigerated box and stored in sealed bags at 0 °C at the laboratory. The water for the experiment was collected at the same site of the sediment using a 20 L bottle and also the physicochemical conditions were registered, however, the water sampling was carried out just a day before the experiment, to minimize any possible change in the water physicochemical characteristics.

## Preparation of laboratory equipment and materials

Before the experiment performing all pieces of equipment were washed and put on an acid solution (HCl 3%) for three days, after that the equipment was dried in a drying oven. The multiparameter Hanna® probe was calibrated for pH and Eh with standard calibration solutions.

• Experiment Laboratory Apparatus Required
• Polypropylene Bottles (1 Liter)
• Plastic Bottles (10 Liters)
• Becker (0,5 Liter)
• Plastic Spatula
• Centrifuge Tubes (60 mL)
• Syringe (50 mL)
• Plastic hose (3 mm diameter)
• Glove bag
• He Gas Cylinder
• Orbital shaker
• Centrifuge (at least 3000 rpm)
• HNO_3_ (concentrated)
• Automatic Pipette
• Milli-Q water
• Analytical Balance

It is important to note, the number of centrifuge tubes should be related to the metal extraction method chosen and the polypropylene bottles and syringes with the number of samples.

## Experiment procedures

**Fig. 1:**
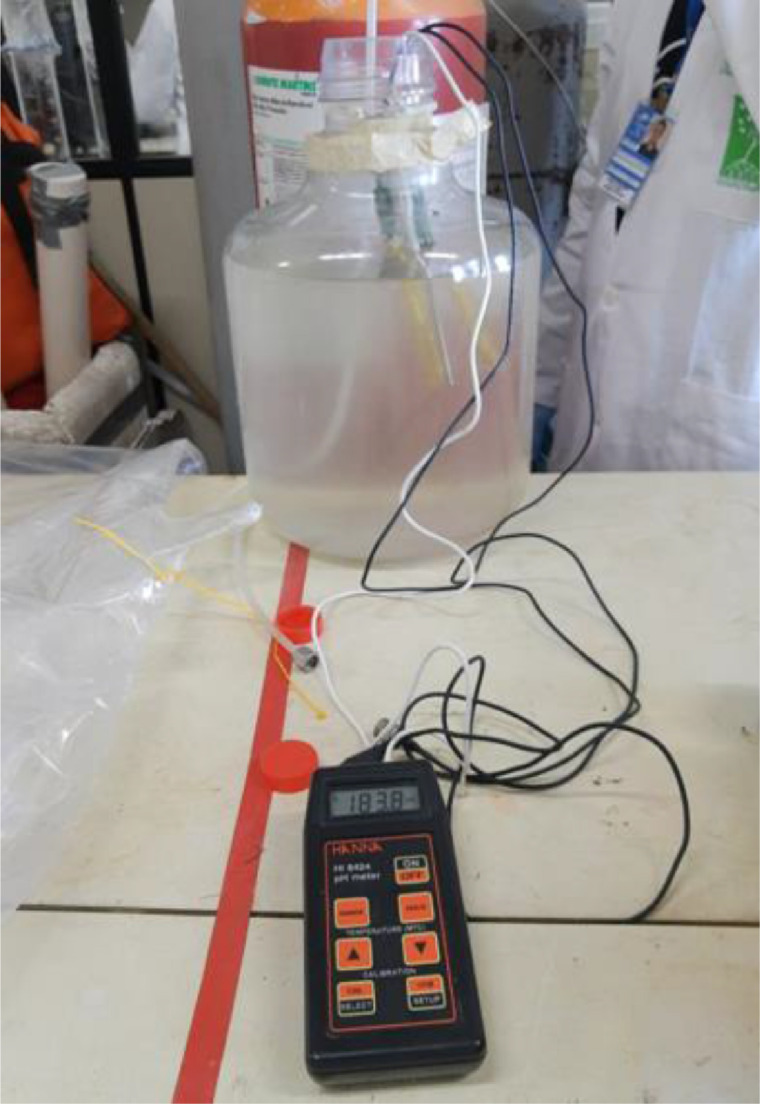
Water dissolved oxygen purging process.

The water preparation for the experiment consists of an attempt to remove as much oxygen dissolved as possible from the water collected on the site. Therefore, the water oxygen purge should be carried out using a He cylinder, for this stick the plastic hose connected with the He gas cylinder, and then let bubble up for at least 30 min (Fig. 1). During the dissolved oxygen purge procedure the Eh and pH should be monitored using an electronic probe.

**Fig. 2:**
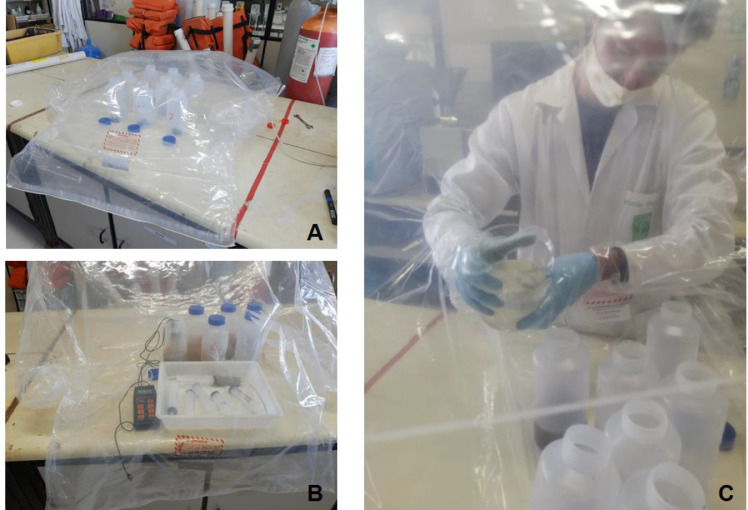
(A) The bottles with sediment positioned inside the glove bag for the experiment beginning. (B) The slurry sampling at the moment T0. (C) The bottles with sediment being carefully filled up with the oxygen purged water.

The sediment/water ratio used for the experiment was 1:2.5, therefore was used 200 g of sediment for 0.5 L of water. The sediment was placed carefully inside the polypropylene bottles using a plastic spatula and weighed in an analytical balance. The bottles with the sediment were placed into an empty glove bag (Fig. 2A), with also the purged water in a 0.5 L becker and the multiparameter probe. At this point, the glove bag should be shut and filled up with He gas, creating an anoxic environment, the experiment manipulation should be made inside de glove bag. The bottles containing the sediment were carefully filled up with purged water (Fig. 2C), after the bottles tightly closed, all experiment bottles were incubated in a dark room for 15 days. Actually the incubation is the main process for generating a severe redox reduction environment, during this period the organic matter content is degraded by bacterial activity. In an environment containing as little dissolved oxygen as possible, the anaerobic bacterial should use other electrons acceptors for organic matter degradation, resulting in the potential redox decrease. The most of trace metals under this condition assumes the reduced specie and tend so bond with other compounds, especially sulfide, tending to stabilize in the sediment reproducing as far as possible the site conditions.

After the incubation period, the bottles were opened inside the glove box filled up with He gas, after a vigorous manual agitation the slurry sampling was carried out using a syringe connected with a plastic hose. This first sample was called T0 and must represent the physicochemical conditions found at the site. For the T1 sampling (Fig. 2B), the bottles were removed from the glove bag and put at an orbital shaker table at 60 rpm, at this moment the oxidation process started, the T1 slurry sample was collected after 10 min of shaking. This sampling procedure was repeated for T2 (120 min shaking), T3 (240 min shaking) and T4 (720 min shaking). For all sampling moments, the Eh and pH were registered using the multiparameter probe. The slurry collected were placed in 60 mL centrifuge tubes and centrifuged for 20 min at 3000 rpm for segregation of suspended particulate matter and supernatant. The particulate matter should be separated of supernatant and lyophilized for further extraction trace metals procedure. The Milli-Q water was used as a blank in the experiment.

## Method validation

**Fig. 3:**
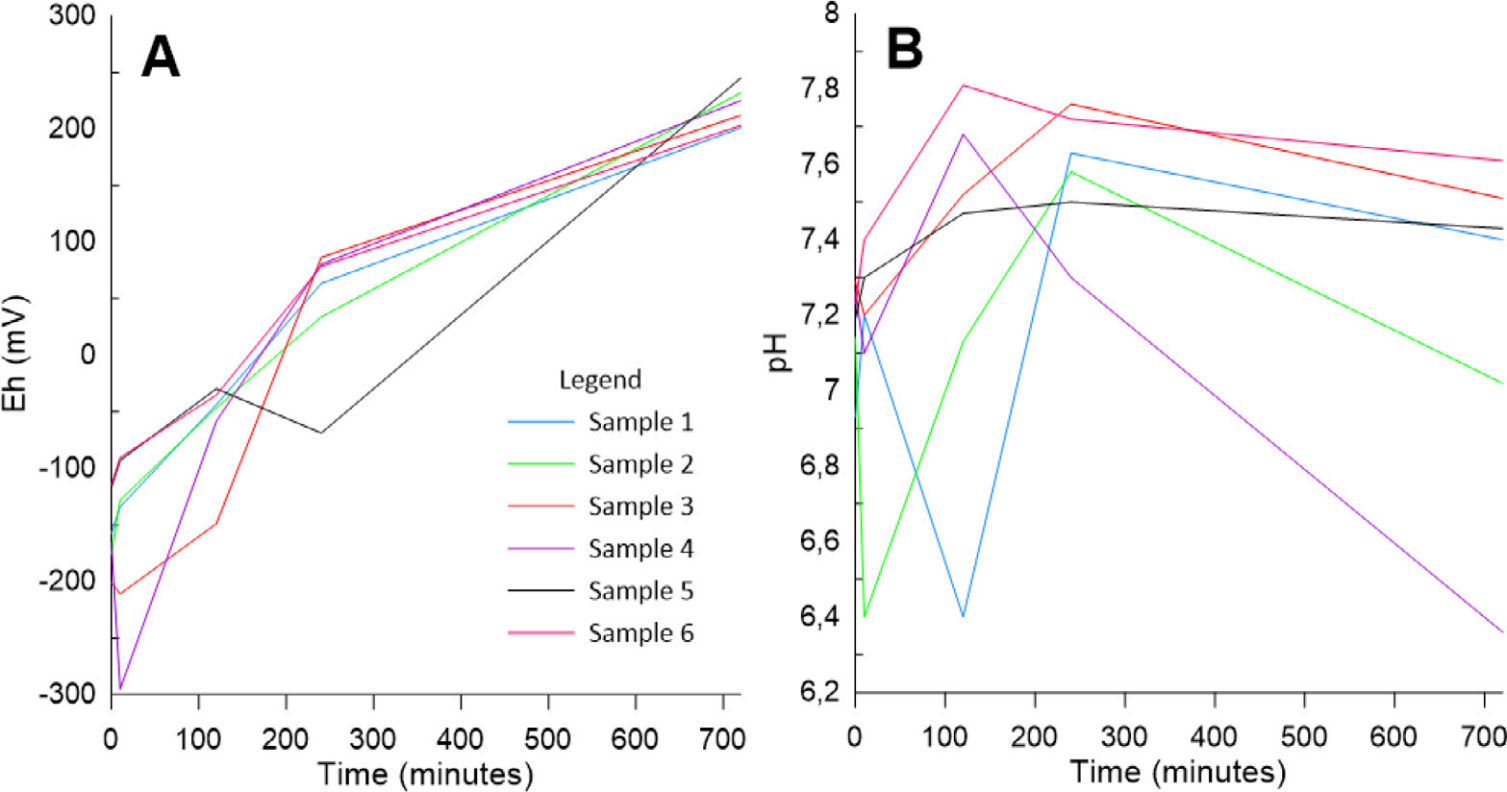
(A) Eh curve during the resuspension experiment and (B) pH curve during the resuspension experiment.

The experiment procedures proved to be successful and simple to simulate changes in potential redox and pH. The 6 samples used in the experiment presented very low potential redox after the incubation period (Fig. 3-A). The oxidation process was also successful for all samples, shaking the slurry at least for 720 min seems to be enough for complete compounds oxidation. A similar pattern was observed in all the 6 sample bottles. Relevant differences among the samples for potential redox could be noted after the incubation period and in the oxidation beginning, however at the end all samples presented similar values. The pH also presented different behavior among the samples, for pH this variation was less relevant, approximately 1 pH unit (Fig. 3-B). The oxidation of reduced compounds tends to decrease the pH and solubilize CaCO_3,_ the HCO_3_ solution input related to CaCO_3_ dissolution contributes to stabilize the pH between 6 and 8 [12]. The sediment sampling was made in different points in the field, furthermore sediment characteristics as grain size and organic matter content may present any difference, reflecting on potential redox and pH variation at the experiment beginning.
